# Morphia and Atropia Hypodermically Administered

**Published:** 1875-02

**Authors:** 


*Morphia and Atropia Hypodermically Administered.*

There has been going the rounds of the medical press a brief note, stating that one-fourth grain of morphia, hypodermically given, is much less apt to be followed by any ill sequences, if given with one-thirtieth grain of atropia. The modifying influence of atropia over morphia is well known; but it is dangerous to habitually give the two agents in the proportion above named. One grain of morphia is supposed to be measurably antagonized by one-twenty-fourth grain of atropia. Hence, the one-fourth grain of morphia can be measurably balanced by one-ninety-sixth grain of atropia. When given in about this proportion, the ill sequential effects of the morphia are often entirely prevented. The dose of one-thirtieth grain of atropia should not be given, except in cases of emergency, as a *nerve stimulant*, as in cases of collapse from cholera, cholera morbus, etc., or when resulting from severe shock. Under any such circumstances, this dose is unrivaled by any other agent, in power to establish a reaction, that will bid fair to be permanent. T. C. S.

